# The prepontine block and its relevance for the development and treatment of hydrocephalus

**DOI:** 10.1007/s00381-024-06323-w

**Published:** 2024-02-20

**Authors:** Carla Richetta, Shelly I. Shiran, Shlomi Constantini, Jonathan Roth

**Affiliations:** 1 Departments of Pediatric Neurosurgery and the Pediatric Brain Center, Dana Children’s Hospital, Tel Aviv Medical Center, 6 Weizman Street, Tel Aviv, 64239 Israel; 2 Pediatric Radiology Unit, Dana Children’s Hospital, Tel Aviv Medical Center, Tel Aviv, Israel; 3 Faculty of Medicine, Tel-Aviv University, Tel-Aviv, Israel

**Keywords:** Prepontine block, Hydrocephalus, Endoscopy

## Abstract

**Objective:**

Pulsatile CSF flow patterns include flow through the ventricles to the subarachnoid space and cisterns and from the infra- to the supratentorial subarachnoid space. In this study, we demonstrate how an obstruction at the level of the prepontine space may lead to obstructive hydrocephalus with specific radiological characteristics, as well as the implications for treatment options.

**Methods:**

We retrospectively collected data of patients who underwent surgery between February 2010 and December 2022 for hydrocephalus secondary to a suspected prepontine block. One additional patient diagnosed with prepontine block who did not undergo surgery was also included. We excluded patients with a background of previous unrelated neurosurgical procedures or CNS infections.

**Results:**

Six children and two adults were included. Three presented with hydrocephalus on imaging, without any other underlying pathology. Five had a suprasellar arachnoid cyst, with its lower border abating the pons and occluding the spinal subarachnoid space (SAS). All cases had an open aqueduct on T2 sagittal sequences, as well as an infracerebellar or retrocerebellar CSF collection. In most cases, a horizontal web was identified in the prepontine region. Seven cases were treated with an endoscopic fenestration. One patient subsequently underwent a shunt surgery. All the operated children reached normal developmental milestones after surgery.

**Conclusions:**

This paper describes a rather small series of cases where clear obstruction was observed at the level of the prepontine subarachnoid space. We believe this anatomical subtlety adds to a better understanding of CSF pathways and the role of ETV in treating hydrocephalus, focusing on a small subgroup of patients without a clear obstruction.

## Introduction

The classic understanding of pulsatile CSF flow is as follows. After its production by the choroid plexus, CSF flows between the ventricles via the foramina of Monro, through the aqueduct of Sylvius, then to egress via foramina of Luschka and Magendie to the cisterns and the spinal subarachnoid space (SAS). CSF then flows from the infratentorial SAS to the supratentorial SAS, to be absorbed by the arachnoid granulations and venous sinuses. Despite several alternative postulations on CSF production by the ependyma and absorption via spinal nerve roots and glymphatic pathways, pulsatile flow is the main basic model for understanding CSF dynamics with relation to surgical treatment options [1]. This flow model serves as a template for surgical decisions, such as who would be a candidate for neuroendoscopy.

However, infra- to supratentorial CSF flow has never been deeply explored. It is generally believed to occur around the brainstem, at the anterior cisterns (prepontine, interpeduncular, suprasellar, chiasmatic), and lateral to the brainstem (crural and ambient). However, several anatomical studies on cadavers have shown that the lateral cisterns (crural and ambient) are often covered by a thick and impermeable arachnoid membrane, separating the supra- and infratentorial compartments. This would lead the main pulsatile flow from the infra- to the supratentorial compartments via the anterior cisterns [2, 3]. It follows that the presence of an obstruction at the level of the prepontine area could impede the CSF from reaching the supratentorial space, potentially resulting in obstructive hydrocephalus.

The concept of prepontine CSF obstruction (prepontine block, PPB) has rarely been described [4, 5]. In their experience, we have found that despite its location outside of the ventricular system, endoscopic third ventriculostomy may often be an effective treatment modality.

In this manuscript, we present several pathologies obstructing the prepontine cistern, leading to extra-ventricular obstructive hydrocephalus. We emphasize the interaction between these pathologies and the CSF physiology and explore the impact on treatment options.

## Methods

Following approval from the Institutional Review Board at the Tel Aviv Medical Center, we performed this retrospective study. The medical records of all consecutive patients between February 2010 and December 2022 with hydrocephalus suspected of being secondary to a PPB were reviewed.

Several radiological findings were required for suspecting a PPB:

- Enlargement of lateral, third, and fourth ventricles
- “Ballooning” of the third ventricle: anterior displacement of the lamina terminalis, downward bowing of the tuber cinereum
- Absence of any intraventricular obstruction: no intraventricular tumor, open (and usually enlarged) aqueduct
- Absence of obstruction at the outlets of the 4th ventricle: good flow void at the Magendie, no outpouching of the Luschka (as would have been expected in fourth ventricle outlet obstruction)
- No leptomeningeal tumor spread

Two types of PPB were defined: primary PPB, a thickened membrane leading to obstructive HCP, and secondary PPB, a block caused by a prepontine pathology other than a spontaneous localized arachnoid membrane. We excluded patients with additional obstructions (e.g., foramina of Monro, aqueduct). Both primary and secondary PPB were included in this case series. There was no age limitation.

Patients were collected from an ongoing department database, after verifying compatibility with the inclusion criteria. Radiological and clinical data were collected from patient electronic files as well as PACS.

Considering the descriptive nature of this paper and the small number of patients, no statistical analysis was completed.

## Results

**Fig. 1:**
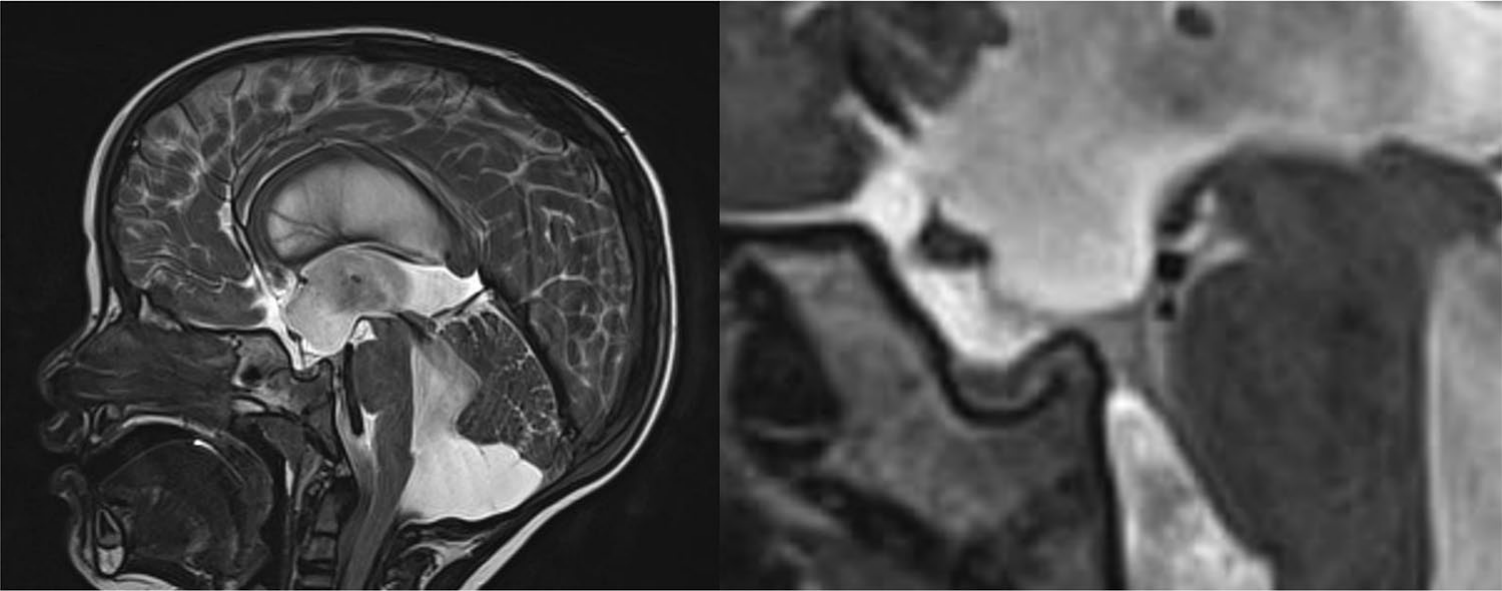
Midsagittal T2 SPACE T2 image showing a flow void through the aqueduct and a “ballooned” third ventricle (left). A slightly parasagittal and zoomed-in cut shows intensity changes in the prepontine region suggesting the presence of a membrane and fluid turbulence in its proximity (primary PPB) (right)

**Fig. 2:**
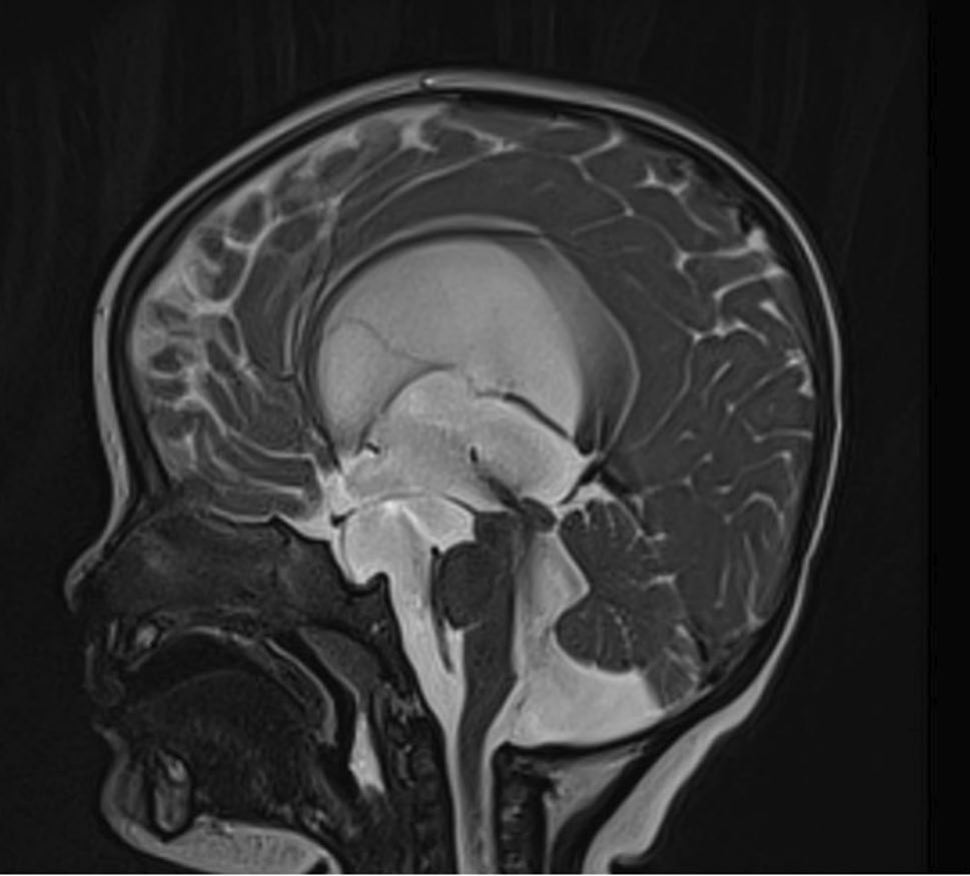
Preoperative MRI of a 1-year-old female showing a suprasellar cyst with a secondary PPB

Over the course of 13 years, eight cases were identified that matched our criteria: two females and six males (Table 1). There were six children, 1–11 years old (3.4 ± 4.3), and two adults, 30 and 33 years old. Three patients had an isolated prepontine membrane (primary PPB) (Fig. 1). Five patients had a suprasellar arachnoid cyst (SSC), with a lower extent to the prepontine cistern (secondary PPB) (Fig. 2). Three patients with suprasellar cysts had a prior shunt.

**Table 1:** Symptoms and signs in pediatric patients

**Patient**	**Age (years)**	**Etiology**	**Prior surgical treatment**	**Presenting symptoms**	**Index treatment**
1	1	SSC		Increased HC	VCC
2	1	SSC		Seizure	VCC
3	1	SSC	VPS	Enlarging cyst, pre-existing shunt	VCC
4	1	SSC	VPS	Enlarging cyst, pre-existing shunt	VCC
5	3	Primary		Increased head circumference and fine motor impairment	ETV
6	11	Primary		Headache	ETV ➜ VPS
7	30	SSC	VPS	Visual decline, pre-existing shunt	VCC
8	33	Primary		Asymptomatic	

*HC* Head circumference, *SSC* Suprasellar cyst, *VCC* Ventriculo-cysto-cisternostomy, *VPS* Ventriculoperitoneal shunt

### Clinical presentation

One adult was asymptomatic at the time of diagnosis and remained asymptomatic during his follow-up (8 months). The other cases had a variety of symptoms (Table 1). Three presented with increased ICP symptoms and signs. Other non-specific symptoms included visual complaints and motor disturbances. The three patients with a pre-existing shunt had presented with an enlarging cyst, two with no new symptoms and one with visual decline.

### Radiological findings

All cases had a posterior fossa CSF collection: seven had an infracerebellar collection, and five had a retrocerebellar collection. One case displayed both collections. In all cases, the fourth ventricle outlet was open.

In all three cases with a primary PPB, a preoperative MRI scan (T2 SPACE sequences) revealed signal changes and anomalies in the CSF flow turbulence in the prepontine region, behaving as though there were both superior and inferior compartments.

In five cases with a suprasellar cyst, the obstruction involved the prepontine region as well as the foramina of Monro and aqueduct.

Third ventricle parameters (third ventricle width and downward bulging of the floor) could not be assessed in some due to anatomical distortion caused by large suprasellar cysts. However, in the five cases where third ventricle width was measurable, the average width was 14.6 mm. Among these cases, four patients displayed downward bulging of the ventricle floor, while in one case, displacement was upward due to the presence of a cyst.

### Treatment

Table 1 summarizes the various treatments: Three patients with SSC had a prior ventriculoperitoneal shunt (VPS). Seven patients in our study group were operated, undergoing an endoscopic procedure. There were two endoscopic third ventriculostomies (ETV) and five endoscopic ventriculo-cysto-cisternostomy (VCC). In both cases with a primary PPB (and no suprasellar cyst) which were operated, a thick prepontine membrane was visualized intraoperatively, which was perforated. There were no intraoperative complications.

### Outcome

Patients were followed clinically and radiologically for an average of 25.2 months since surgery (range 2–73 months).

There were no new neurological deficits following surgery. All pediatric patients reached normal developmental milestones. At time of last follow-up, seven patients were asymptomatic, and one had headaches. One patient with primary PPB underwent insertion of a ventriculoperitoneal shunt (VPS), due to lack of headache improvement, and continues to suffer from headaches.

## Discussion

Prepontine block (PPB) as a cause for obstructive hydrocephalus is rarely diagnosed and discussed. In this small series, we present our experience with both primary PPB and secondary PPB with suprasellar cysts. The “tentorial block” at the crural and ambient cisterns, as described by anatomical dissections [2, 3], leads to pulsatile CSF flow exclusively anterior to the brainstem. This notion is not commonly known, but it explains how a PPB may lead to obstructive hydrocephalus.

The radiological diagnosis of PPB is not trivial and necessitates both suspecting the condition and looking for fine radiological nuances. Hydrocephalus with a “ballooned” third ventricle is a common finding in primary PPB (low lying 3rd ventricular floor, anterior bulging lamina terminalis), as well as open aqueduct and a “flow cut” anterior to the pons, as seen on flow sensitive MRI sequences (e.g., cine MRI, T2 SPACE). In secondary PPB, a local pathology (such as a large suprasellar cyst) with a low-lying inferior membrane suggests a possible local mechanism. When in doubt, MRI or CT cisternography or ventriculography may identify the location of an obstruction [4, 5]. High-resolution MRI sequences (such as FIESTA or CISS) may also show local adhesions in the prepontine cistern.

As shown in our small series, for both primary and secondary PPB, all cases were associated with retro-/infracerebellar CSF collections. Infracerebellar CSF collections do have differential diagnoses: mega cisterna magna, Blake’s pouch, and arachnoid cysts. The last two are sometimes associated with hydrocephalus. Differentiation between these options may necessitate a CT or MRI contrast cisternography [4, 5]. We presume that in PPB caused by various congenital causes (such as primary PPB and PPB caused by suprasellar cysts), the infra-/retrocerebellar CSF collections are a “mega cisterna magna,” serving as a CSF “buffer” proximal to the obstruction. Thus, in the presence of hydrocephalus in association with an infra-/retrocerebellar CSF collections, PPB should be included in the differential diagnosis, with the corresponding treatment implications.

In primary PPB, performing an ETV may resolve the obstruction, similar to the role of ETV in fourth ventricle outlet obstruction (FVOO) [6]. Currently, we have found only one series of patients with a PPB who underwent an ETV [7]. In this series of 21 pediatric patients with characteristic radiological findings suggesting an obstruction in the prepontine area, 15 patients were able to avoid a VPS following ETV. Failures were observed only in patients younger than 1 year of age. Technically, there is no need to perforate the obstructing membrane, as the ETV will bypass the obstruction, communicating the ventricles to the interpeduncular cistern.

Secondary PPB may occur in the context of a suprasellar arachnoid cyst (SSC), as well as other local obstructing pathologies, such as tumors. Suprasellar arachnoid cysts arise from the diencephalic part of the Liliequist membrane [8–10]. SSC may cause hydrocephalus due to an obstruction at one of several locations. For example, large cysts that elevate the third ventricular floor may lead to obstruction of the foramina of Monro. A posterior compression will lead to an obstruction of the aqueduct. However, even in the absence of these obstructions, the inferior part of the cyst may cause a PPB in a manner similar to that of the primary PPB described above. Treatment of SSC includes a ventriculo-cysto-cisternostomy (VCC) [11–13]. A VCC will reduce the cyst volume and resolve the obstructing cause in cases obstructing the foramina of Monro, as well as reduce the aqueductal compression in those with a significant posterior compression. For the PPB, a reduced mass effect from the cyst will possibly elevate the lower obstructing membrane, alleviating the obstruction. Additionally, fenestration of the prepontine membrane will enable CSF to flow from the upper to the lower side, dissecting the route to the normal distal cisterns (e.g., chiasmatic and carotid). It is important to state that VCC enjoys an 80–90% success rate [11–13]. A possible explanation for failure of VCC may be obstruction of the pathways to the chiasmatic and carotid cisterns. This may be overcome either by a lamina terminal ostomy or a shunt.

## Limitations

As this is a retrospective study, and given that there is no gold standard diagnostic criteria for PPB, instances of PPB diagnosis may have been overlooked and not included in this series. For example, if patients did not undergo CT or MR cisternography, PPB might not have been identified and thus, the diagnosis may have been inaccurate.

## Conclusions

Prepontine block is an under-recognized etiology for obstructive hydrocephalus. These include both primary and secondary PPB, such as cases with suprasellar cysts. We have shown an association between PPB and retro/infracerebellar CSF collections. Treatment options may include an ETV, especially in the non-infantile age group.

## Data Availability

No datasets were generated or analyzed during the current study.
